# FKBPL Is a Critical Antiangiogenic Regulator of Developmental and Pathological Angiogenesis

**DOI:** 10.1161/ATVBAHA.114.304539

**Published:** 2015-03-25

**Authors:** Anita Yakkundi, Rachel Bennett, Ivette Hernández-Negrete, Jean-Marie Delalande, Mary Hanna, Oksana Lyubomska, Kenneth Arthur, Amy Short, Hayley McKeen, Laura Nelson, Cian M. McCrudden, Ross McNally, Lana McClements, Helen O. McCarthy, Alan J. Burns, Roy Bicknell, Adrien Kissenpfennig, Tracy Robson

**Affiliations:** From the McClay Research Centre for Pharmaceutical Sciences, School of Pharmacy (A.Y., R.B., M.H., O.L., A.S., H.M., L.N., C.M.M., R.M., L.M., H.O.M., T.R.), Centre for Infection and Immunity (M.H., O.L., A.K.), and Northern Ireland Molecular Pathology Laboratory, Centre for Cancer Research and Cell Biology (K.A.), School of Medicine, Dentistry and Biomedical Sciences, Queen’s University, Belfast, UK; School of Immunity and Infection and Cancer Studies, Institute for Biomedical Research, University of Birmingham, Birmingham, UK (I.H.-N., R.B.); Centre for Digestive Diseases, Queen Mary, University of London, Barts and The London School of Medicine and Dentistry, London, UK (J.-M.D.); and Birth Defects Research Centre, UCL Institute of Child Health, London, UK (J.-M.D., A.J.B.).

**Keywords:** angiogenesis, CD44, FKBPL, knockout, vasculature

## Abstract

**Objective—:**

The antitumor effects of FK506-binding protein like (FKBPL) and its extracellular role in angiogenesis are well characterized; however, its role in physiological/developmental angiogenesis and the effect of *FKBPL* ablation has not been evaluated. This is important as effects of some angiogenic proteins are dosage dependent. Here we evaluate the regulation of FKBPL secretion under angiogenic stimuli, as well as the effect of *FKBPL* ablation in angiogenesis using mouse and zebrafish models.

**Approach and Results—:**

FKBPL is secreted maximally by human microvascular endothelial cells and fibroblasts, and this was specifically downregulated by proangiogenic hypoxic signals, but not by the angiogenic cytokines, VEGF or IL8. FKBPL’s critical role in angiogenesis was supported by our inability to generate an *Fkbpl* knockout mouse, with embryonic lethality occurring before E8.5. However, whilst *Fkbpl* heterozygotic embryos showed some vasculature irregularities, the mice developed normally. In murine angiogenesis models, including the ex vivo aortic ring assay, in vivo sponge assay, and tumor growth assay, *Fkbpl*^+/−^ mice exhibited increased sprouting, enhanced vessel recruitment, and faster tumor growth, respectively, supporting the antiangiogenic function of FKBPL. In zebrafish, knockdown of zFkbpl using morpholinos disrupted the vasculature, and the phenotype was rescued with h*FKBPL*. Interestingly, this vessel disruption was ineffective when *zcd44* was knocked-down, supporting the dependency of zFkbpl on zCd44 in zebrafish.

**Conclusions—:**

FKBPL is an important regulator of angiogenesis, having an essential role in murine and zebrafish blood vessel development. Mouse models of angiogenesis demonstrated a proangiogenic phenotype in *Fkbpl* heterozygotes.

## Abstract 2

Supplemental Digital Content is available in the text.

The vascular system fulfils the oxygen and nutrient requirements of tissues and organs, whilst maintaining tissue growth and repair. Vasculogenesis is a highly orchestrated process in embryonic development where de novo vessel formation takes place from angioblasts, the endothelial precursor cells.^1^ Subsequent sprouting from existing vessels, that is angiogenesis, leads to a vascular network of arteries, veins, and capillaries. In adult organisms, this process is quiescent; however, endothelial cells retain their capacity to differentiate into new vessels in response to angiogenic signals when required. Angiogenesis is deregulated in disease states; it is inadequate in diseases, such as stroke, ischemia, and diabetic wound healing^2^ and excessive in cancer, inflammatory disorders, and eye diseases,^3,4^ thus, offering tremendous scope for therapeutic intervention.^4^ Many pro- and antiangiogenic strategies have been developed; although the proangiogenic approaches have not been successful to date, antiangiogenic therapies have been moderately effective in the treatment of cancer and eye diseases.^5^ Clinically approved antiangiogenic anticancer agents primarily target the VEGF pathway and have many limitations, such as increased resistance, toxicity,^6^ secondary metastases,^7^ and limited efficacy in certain cancer types, highlighting the need for novel agents targeting alternative pathways.

FK506-binding protein like (FKBPL), a novel member of the immunophilin protein family,^8,9^ is a potent secreted antiangiogenic protein^10,11^ targeting the CD44 pathway.^12^ FKBPL is also a prognostic biomarker for breast cancer and predictive for endocrine therapy.^13,14^ Therapeutic peptide derivatives of FKBPL, AD-01 and ALM201, have highly potent antiangiogenic activity, and because of their CD44-mediated effect, these agents are also able to target CD44-positive cancer stem cells,^15^ further strengthening their therapeutic potential. Following extensive preclinical validation, ALM201 has now completed toxicological evaluation in preparation for phase I clinical trials in cancer patients. Although the robust antiangiogenic effects of FKBPL and its therapeutics have been well characterized, the role of endogenous FKBPL in physiological and developmental angiogenesis is not. Here we address these questions using cell lines, knockout (KO) mice, and zebrafish models. We have developed an *Fkbpl* heterozygote mouse model and characterized the functional role of FKBPL in a range of in vivo and ex vivo angiogenesis end points. Furthermore, we have used GFP-zebrafish that provides an easy and strong model for evaluating developmental angiogenesis.^16^ We report that the antiangiogenic protein, FKBPL, plays a critical role in embryonic development, and its secretion is regulated by hypoxia. *Fkbpl* heterozygous mice upregulated angiogenesis in all models evaluated, strengthening the biological role of FKBPL in angiogenesis and supports FKBPL-based diagnostic and therapeutic interventions as they advance to clinical trials.

## Materials and Methods

Materials and methods are available in the online-only Data Supplement.

## Results

### FKBPL Secretion Is Regulated by Hypoxia

**Figure 1.**
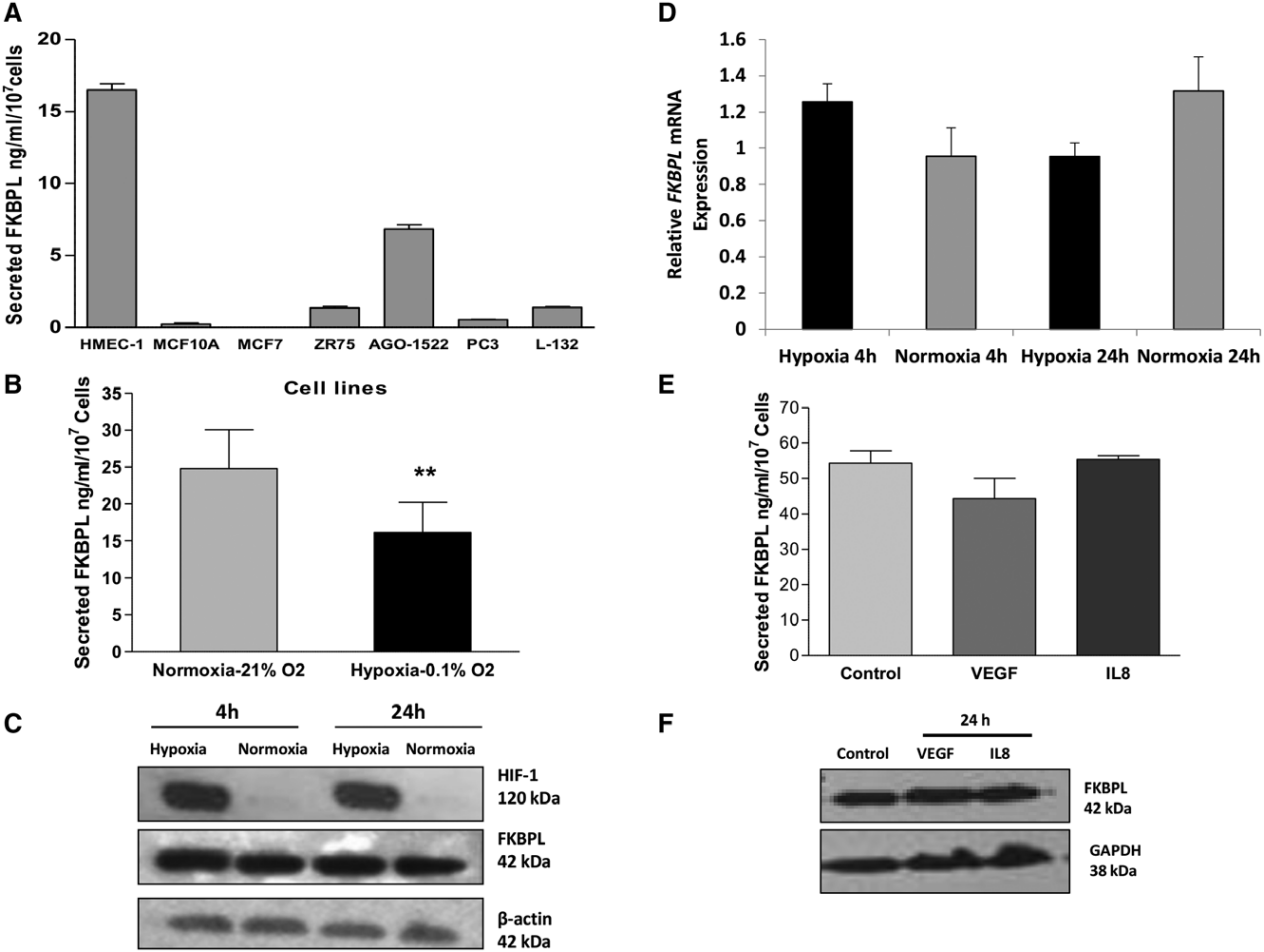
Regulation of FK506-binding protein like (FKBPL) secretion. **A**, ELISA demonstrating that FKBPL was maximally secreted by endothelial (HMEC-1) and fibroblast (AGO-1522) cell lines, in comparison to tumor cell lines, and **B**, its secretion was specifically downregulated under hypoxia in HMEC-1 cells; intracellular protein (**C**) or mRNA (**D**) levels assessed by qRT-PCR did not change. Proangiogenic cytokines, VEGF, 25 ng/mL, or IL8, 1 nmol/L, did not alter FKBPL secretion (**E**) or intracellular (**F**) levels. A log phase 24 h old monolayer was subjected to different oxygen tensions or cytokine treatments for 24 h and the spent medium; RNA and protein from cell lysates were harvested. FKBPL levels were quantified by ELISA of 50-fold concentrated spent medium and normalized to cell number at the time of harvest. Data points are mean±SEM. n≥3. **P<0.01 paired *t* test.

We have previously reported that extracellular FKBPL exerts its antimigratory effects via CD44, and this effect can be reversed using a blocking antibody specific to the active angiogenic site of FKBPL.^12^ When secreted levels of FKBPL from various cell lines were evaluated by ELISA, maximal secretion (15–20 ng/10^7^ cells and ≈7 ng/10^7^ cells) was observed in endothelial cells, human microvascular endothelial cells (HMEC-1), and normal human fibroblasts, AGO-1552, respectively. Cancer cell lines or the normal breast epithelial cell line, MCF10A, secreted lower levels, ≤1.5 ng/10^7^ cells (Figure 1A). The secretion from HMEC-1 was specifically inhibited when cells were cultivated in a proangiogenic hypoxic environment (0.1% O_2_) for 24 h (Figure 1B), whereas *FKBPL* mRNA or intracellular FKBPL protein levels remained unchanged (Figure 1C and 1D). HIF-1 increased upon hypoxic stimulation, acting as a positive control. Other proangiogenic stimulators, such as VEGF, IL-8, and bFGF, did not significantly alter FKBPL secretion, protein or mRNA expression (Figure 1E and 1F; Figure I in the online-only Data Supplement). Having established that FKBPL secretion was inhibited by proangiogenic hypoxic signaling, we proceeded to evaluate its endogenous role in development and pathological angiogenesis using knockout models.

### Fkbpl Heterozygous Knockout Mice Appear Normal but Homozygous Knockout Is Embryonically Lethal

**Figure 2.**
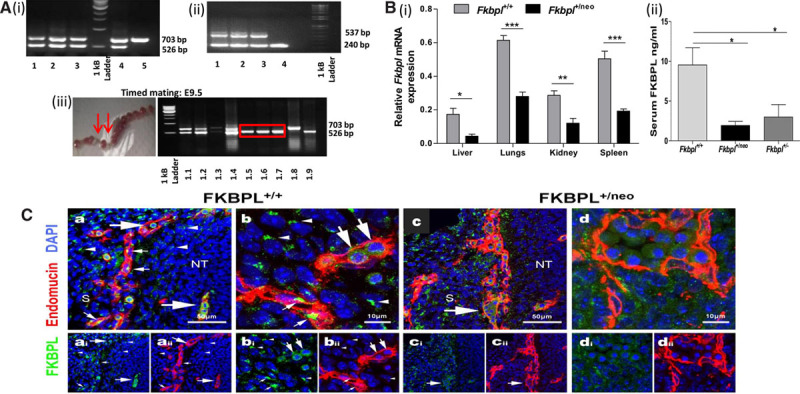
*Fkbpl* knockout mice and embryo characterization. **A**, Genomic PCR of (**i**) *Fkpbl*^+/neo^ (lanes 1–4) heterozygotes and *Fkpbl*^+/+^ litermate controls (lane 5) and (**ii**) *Fkpbl*^+/−^ (lanes 1–3) and *Fkpbl*^+/+^ (lane 4) genotypes. (**iii**) Timed matings of the *Fkpbl* heterozygous crosses, demonstrating E9.5 embryos showing empty or resorbed yolk sac (*arrows*) and genotyping of the embryonic yolk sac; *Fkpbl*^+/+^ (lane 1.8), *Fkpbl*^+/neo^ (lanes 1.1–1.4, 1.9) and *Fkpbl*^neo/neo^ (lanes 1.5–1.7; boxed) alleles. Homozygous *Fkpbl*^neo/neo^ knockout corresponded to resorbed embryos. **B**, FK506-binding protein like (FKBPL) expression in adult mice (**i**) real-time quantitative PCR analysis of RNA from tissue lysates of murine organs, validating the effect of genomic knockdown on *Fkbpl* mRNA and (**ii**) ELISA of serum for secreted FKBPL; reduced levels in *Fkbpl*^+/neo^ and *Fkbpl*^+/−^ mice in comparison to their *Fkbpl*^+/+^ littermates. Murine organs were harvested in RNA*late*r, followed by RNA extraction qPCR quantification. Serum was collected from age-matched mice bleeds. Data points are mean±SEM. n≥3. *P<0.05, **P<0.01, ***P<0.001 (B(i) unpaired *t*-tests and B(ii) 1-way ANOVA, Tukey multiple comparison). **C**, Expression of FKBPL and endomucin in intersomitic vessels of *Fkbpl*^+/+^ and *Fkbpl*^+/neo^ mice at E11.5. Transverse sections at the level of an intersomitic vessel showing merged images of FKBPL expression in *Fkbpl*^+/+^ embryo (**a**, 40×; **b**, 63×, magnification), single channel (**ai, bi**) FKBPL (green) and (**aii, bii**) endomucin (red). FKBPL is strongly expressed in endomucin^+ve^ cells (yellow merge; small arrows) and in nucleated rounded cells, in the lumen of blood vessels (big arrows). Intersomitic vessels show stereotypical pattern. Merged images (c, d) showing reduced FKBPL expression in a cluster of round nucleated cells located within the lumen of an endomucin^+ve^ intersomitic blood vessel in *Fkbpl*^+/neo^ mice. Intersomitic vessels show normal pattern but seem locally distended by cluster of intraluminal cells (big arrow). Single channel images: (**ci**) FKBPL (green) and (**cii**) endomucin (red). All slides were counterstained with DAPI (blue). Scale bar=50 μm in **a** and **c**, 40× and 10 μm in **b** and **d**, 63×.

The *Fkbpl*-targeted allele was deleted in the exon 2 region (Figure IIAi and IIAii in the online-only Data Supplement) and following germline transmission resulting progeny were genotyped; the presence of one band at 744 bp corresponded to *Fkbpl* wildtype allele and an additional band at 526 bp corresponded to the *Fkbpl*-targeted allele (Figure 2Ai). Intercrossing of *Fkbpl*^+/neo^ mice did not yield any *Fkbpl*^neo/neo^ mice, with over 50 progeny, indicating that the homozygous KO of *Fkbpl* was embryonically lethal. The *Fkbpl*^+/neo^ mice retained the neomycin selective marker; therefore, to exclude any interference from expression of this marker, it was deleted using CRE^tg/+^ mice with 2 sequential crosses with *Fkbpl*^+/neo^ mice to obtain *Fkbpl*^+/−^ mice (Figure IIAiii in the online-only Data Supplement) that were genotyped to confirm the presence of 2 bands at 537 and 240 bp for *Fkbpl*^+/−^ mice and a single band at 240 bp identifying the *Fkbpl*^+/+^ mice (Figure 2Aii). Subsequently, embryos from timed matings were analyzed at E8.5-E13. The presence of empty yolk sacs and resorbed embryos was observed at E8.5 and E9.5 (Figure 2Aiii), suggesting lethality within the *Fkbpl*^neo/neo^ line, confirmed by genotyping of the yolksacs (Figure 2Aiii); intact *Fkbpl*^+/neo^ embryos did not demonstrate any gross phenotypic changes. Embryonic lethality was also confirmed in the *Fkbpl*^−/−^ mice because of the inability to obtain *Fkbpl*^−/−^ mice with several (>50) intercrosses of *Fkbpl*^+/−^ mice.

*Fkbpl*^+/neo^ mice were then further characterized. Genomic KO of one *Fkbpl* allele resulted in ≈2-fold reduction of *Fkbpl* mRNA in all organs analyzed (Figure 2Bi). Reduced levels of *Fkbpl* expression because of loss of one allele was also confirmed in the *Fkbpl*^+/−^ mice (Figure IIC in the online-only Data Supplement). However, serum FKBPL levels were downregulated up to 4-fold in the *Fkbpl*^+/neo^ or *Fkbpl*^+/−^ mice in comparison to *Fkbpl*^+/+^ littermates (Figure 2Bii); there was no significant difference in FKBPL levels between *Fkbpl*^+/neo^ or *Fkbpl*^+/−^ mice. The *Fkbpl*^+/neo^ and *Fkbpl*^+/−^ mice developed normally with no overt abnormalities, and the organ histology appeared normal (Figure IIB in the online-only Data Supplement).

### Embryonic Expression of FKBPL and Effects on Vasculature

During early development (E11.5), FKBPL was strongly expressed in endothelial (endomucin+) cells of the blood vessels, as shown by yellow (red–green merged) staining (Figure 2Ca and 2Cb, small arrows), consistent with Figure 1A. FKBPL was also expressed in nucleated rounded cells located within blood vessels (Figure 2Ca and 2Cb, big arrows; video in the online-only Data Supplement). Additionally, FKBPL was expressed in many other cell types, as an intense subcellular spot, closely associated with the nucleus (Figure 2Ca and 2Cb, small arrows). In accordance with the qPCR data presented in Figure 2Bi, heterozygote mice showed reduced FKBPL immunostaining (Figure 2Cc and 2Cd). Interestingly, accumulation of rounded FKBPL^+^ cells was often seen within the blood vessels of *Fkbpl*^+/neo^ embryos (Figure 2Cc and 2Cd, big arrow). However, this did not affect the overall development of the *Fkbpl*^+/neo^ mice because the new born and adult mice appeared normal.

### Increased Ex Vivo Sprouting of Aortae in Fkbpl Heterozygotic Mice

**Figure 3.**
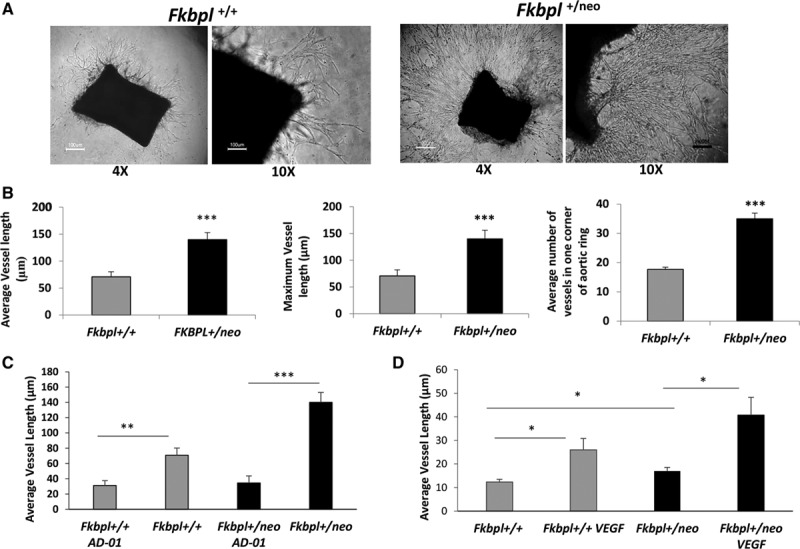
FK506-binding protein like (FKBPL) regulates ex vivo sprouting of vessels from mouse aorta. Representative photomicrographs (**A**) and vessel measurements (**B**) of aortic rings showing enhanced sprouting of vessels from the *Fkbpl*^+/neo^ aortae in comparison to *Fkbpl*^+/+^ aortae, **C**, Inhibition of sprouting mediated by AD-01 (100 nmol/L). **D**, Additive effect of VEGF (30 ng/mL) treatment on vessel sprouting in *Fkbpl*^+/+^ and *Fkbpl*^+/neo^ aortae. Sliced, excised aortae were embedded into matrigel±AD-01 or VEGF and vessel sprouting evaluated microscopically after 10 days. Data points are mean±SEM. n≥3. *P<0.05, ***P<0.001 (1-way ANOVA, C, D; Tukey multiple comparison post tests).

Further characterization of *Fkbpl* KO in angiogenesis was performed by assessing the effect on aortic sprouting ex vivo. The aortae from *Fkbpl*^+/neo^ mice showed enhanced sprouting and disordered branching (Figure 3A). Quantification revealed a statistically significant increase (≈2 fold) in vessel branching and vessel length (Figure 3B). The endothelial origin of the sprouts was confirmed by staining aortic rings with endomucin (Figure IV in the online-only Data Supplement). The *Fkbpl*^+/+^ littermates showed an identical phenotype in the aortic sprouting to that of WT C57Bl6 mice as expected (Figure IIIA in the online-only Data Supplement). *Fkbpl*^+/−^ aortae showed an enhanced pattern of sprouting identical to *Fkbpl*^+/neo^ aortae (Figure IIIB in the online-only Data Supplement), indicating that the 2 murine *Fkbpl*^+/neo^ and *Fkbpl*^+/−^ strains have a similar phenotype. To demonstrate that this was caused by a reduction in the levels of the antiangiogenic protein, FKBPL, AD-01, the therapeutic peptide derived from the antiangiogenic domain of FKBPL, was added to the assay conditions. As expected, this abrogated the enhanced sprouting observed in *Fkbpl*^+/neo^ aortae because of restoration of the FKBPL antiangiogenic function by complementing the activity levels. The angiogenic activity of the *Fkbpl*^+/+^ aortae was also significantly inhibited as expected (Figure 3C). Treatment of the aortic rings with VEGF enhanced the sprouting of *Fkbpl*^+/+^ aortae and had an additive enhancement effect on *Fkbpl*^+/neo^ aortae (Figure 3D), suggesting that FKBPL-mediated sprouting is independent of VEGF angiogenesis pathway.

### Fkbpl Heterozygous Mice Support Enhanced Vessel Recruitment In Vivo in the Sponge Assay

**Figure 4.**
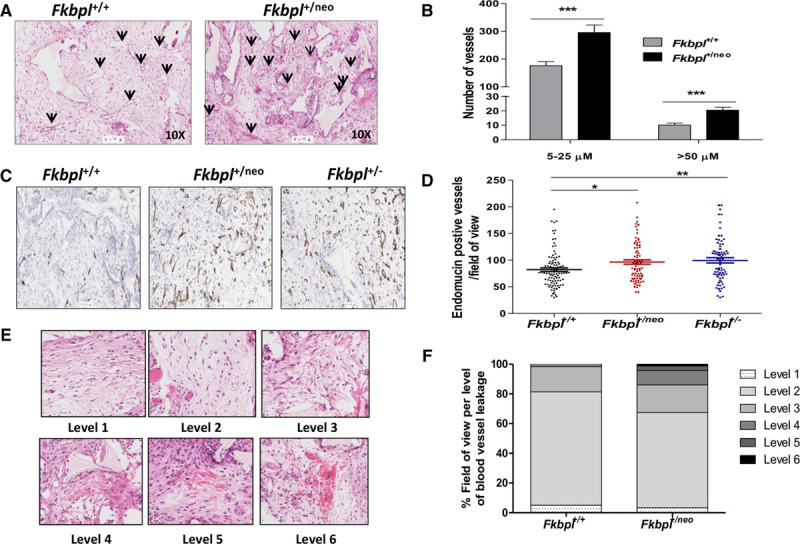
Increased vessel recruitment and vessel leakage in polyether sponges implanted in *Fkbpl*^+/neo^ mice in comparison to *Fkbpl*^+/+^ littermates. Demonstrative H&E images of sponge sections; arrows highlighting the vessels (**A**), vessel quantification on H&E stained sponge sections (**B**); representative images of endomucin stained (brown) sponge sections (**C**); quantification of endomucin stained vessels in sponge sections (**D**); images showing the different levels of blood leakage in H&E stained sections (**E**); quantification of blood leakage in H&E stained sections (**F**). Subcutaneously implanted polyether sponges were treated with murine bFGF for 15 days, excised, formalin fixed, paraffin embedded, and stained with H&E or antiendomucin antibody. Data points are mean±SEM. n≥3. *P<0.05, **P<0.01, ***P<0.001 (B, unpaired *t* test; D, 1-way ANOVA, Tukey multiple comparison post tests).

The effect of *Fkbpl* ablation was further assessed in vivo in a sponge assay. Polyether sponges were implanted into *Fkbpl*^+/neo^, *Fkbpl*^+/−^, and *Fkbpl*^+/+^ mice, and microvessel recruitment was stimulated with bFGF. H&E stained sections of sponges from *Fkbpl*^+/neo^ mice had a significantly higher number of small (40%) and big (50%) vessels (Figures 4A and 4B). The endothelial specificity of this effect was further confirmed using endomucin staining; a significantly higher number (15% to 20%) of endomucin-stained vessels were observed in sponges from *Fkbpl*^+/neo^ (*P*<0.05) and *Fkbpl*^+/−^ (*P*<0.005) in comparison to the *Fkbpl*^+/+^ littermates. This enhanced angiogenesis, however, also corresponded with an increase in the number of erythrocytes leaking into the surrounding tissue (Figures 4E and 4F), indicating that vessels within FKBPL-deficient mice may be less robust.

### Higher Tumor Growth Rate and Tumor Angiogenesis in Fkbpl Heterozygous Mice

**Figure 5.**
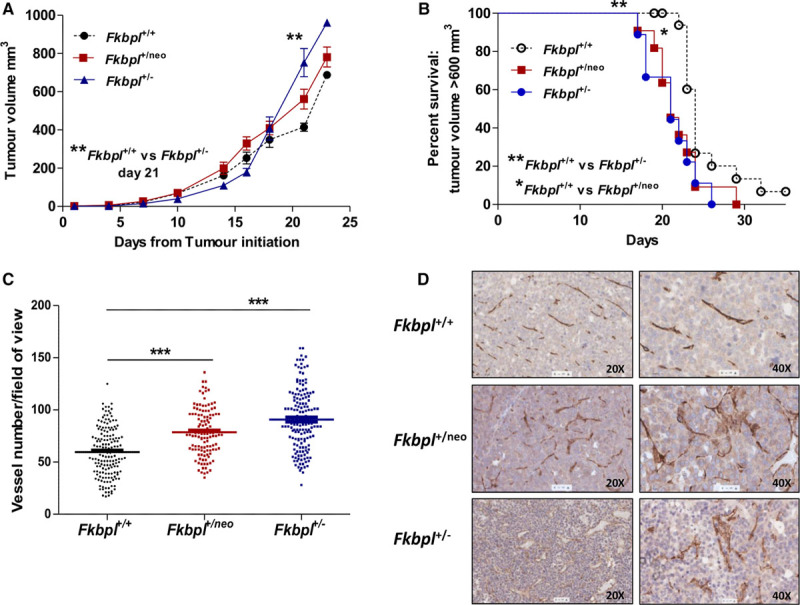
Lewis lung carcinoma tumor implanted in *Fkbpl* heterozygous mice demonstrated increased growth rate and angiogenesis in comparison to tumors grown in *Fkbpl*^+/+^ mice. Growth rate of tumors (**A**) and survival curves (**B**), defined as tumors >600 mm^3^ implanted in *Fkbpl*^+/+^, *Fkbpl*^+/neo^, *Fkbpl*^+/−^ mice. Quantification of vessels numbers (**C**) and photomicrographs of images of CD31-stained tumors sections (**D**). Lewis lung carcinoma (LLC) tumors were intradermally implanted in the mice and tumor volume measured every 2 days. Tumors were excised ≥600 mm^3^, formalin-fixed, and subjected to immunohistochemistry for CD31. Data points are mean±SEM. n≥3. *P<0.05, **P<0.01, ***P<0.001 (1-way ANOVA, Tukey multiple comparison post tests).

Role of *Fkbpl* ablation in pathological angiogenesis was evaluated in the syngeneic Lewis lung carcinoma tumor model. Lewis lung carcinoma cells grown on the rear dorsum of *Fkbpl*^+/neo^ or *Fkbpl*^+/−^ mice had an increased growth rate in comparison to the *Fkbpl*^+/+^ littermates (Figure 5A), corresponding to a significantly shorter survival, defined as time to reach >600 mm^3^ in tumor volume (Figure 5B) in the *Fkbpl* heterozygotes. Immunohistology of the tumors for CD31 expression was performed to assess the effect on tumor vasculature; an increase in CD31-stained blood vessels (Figure 5C) was observed in *Fkbpl*^+/neo^ and *Fkbpl*^+/−^ mice. Gross examination of the blood vessels suggested an irregular structure, with the endothelium appearing thicker and less organized in tumors grown in *Fkbpl*^+/neo^ and *Fkbpl*^+/−^ mice in comparison to a well-defined and ordered vasculature in *Fkbpl*^+/+^ littermates (Figure 5D): a feature of enhanced angiogenesis.

### Fkbpl Regulates Developmental Angiogenesis in the Zebrafish

Having established that KO of one allele of *Fkbpl* produced a proangiogenic phenotype in a range of murine assays, we proceeded to evaluate this effect in zebrafish. NCBI Standard Protein Blast analysis identified Similar to WAF-1/CIP1 stabilizing protein 39 isoform 2 (GenBank ID: XP_001923918.1) as the zebrafish orthologue to the human FKBPL protein (GenBank ID: NP_071393.2). ClustalW2 alignment showed 27% identity between the 2 sequences (Figure VA in the online-only Data Supplement). Analysis of the protein domains using SMART (Simple Modular Architecture Research Tool) showed a similar structure in the C-terminal tetra-trico peptide repeat (TPR) domains, typical of almost all FKBP family members (Figure VB in the online-only Data Supplement).

**Figure 6.**
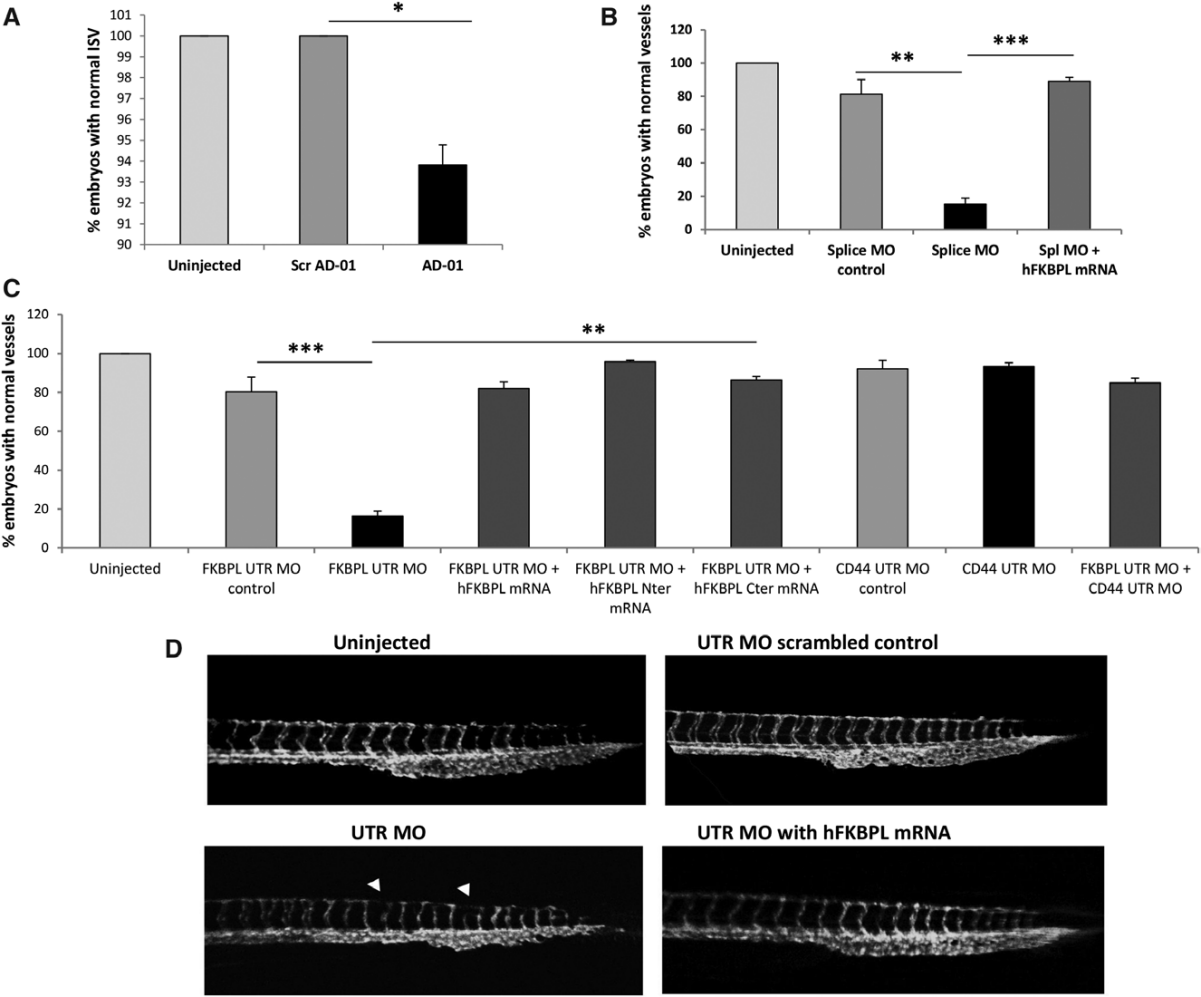
FK506-binding protein like (FKBPL) regulates vessel development in zebrafish embryos (**A**). Vessel quantification of 72 hpf embryos microinjected with AD-01 (1.3 pmol), demonstrating the inhibition of the intersegmental vessel (ISV) development. **B** and **C**, Graphs demonstrating a significant reduction in the number of embryos with normal vessels after injection of the Splice MO (**B**) and UTR MO (**C**), and significant phenotype rescue after coinjection of full length, N or C terminal h*FKBPL* mRNA in a Cd44-dependent manner. Embryos showing intact ISV formation at the indicated time points are defined as normal embryos in the quantification. Data points are mean±SEM. n≥3. *P<0.05, **P<0.01, ***P<0.001 (1-way ANOVA, Tukey multiple comparison post tests). **D**, Photomicrographs of 48 hpf embryos injected with control MO/*zfkbpl* MO (UTR) showing the disrupted vasculature; arrows indicate the trunk and tail vessels, and this was rescued by expression of full length *hFKBPL* mRNA. All the images are lateral views, anterior toward left.

Microinjection of AD-01 into zebrafish embryos at 72 hpf inhibited the intersegmental vessels (Figure 6A), whereas scrambled AD-01 did not affect the vasculature and showed a similar pattern to uninjected embryos. Vessel abnormalities were seen in ≈10% embryos. However, this effect was statistically significant (*P*<0.05); 400 embryos were assessed to verify the effect. This demonstrated the effectiveness of the FKBPL antiangiogenic activity across species.

We also investigated the function of *zfkbpl* during zebrafish embryonic development by microinjection of *zfkbpl*-targeted morpholinos (MO). Injection of splice and translation blocking MOs (UTR MO), leading to formation of a nonfunctional Fkbpl protein impaired the formation of the dorsal longitudinal anastomotic vessels and intersegmental vessel in ≈85% of the injected embryos at 48 hpf (Figure 6B and 6C; *P*=0.006 for UTR MO and *P*=0.01 for splice MO). Interestingly, this also reduced the number of vessels that form the caudal vein, making it look thinner. This vessel inhibition with *zfkbpl* knockdown is contrary to the earlier findings of enhanced angiogenesis with loss of one allele in our mouse model and could possibly be as a result of levels of Fkbpl. Conditional *Fkbpl*^−/−^ KO models could help further identify any possible dosage-related effects that FKBPL might have. Notably, the UTR control MO and splice control MO did not alter the vasculature significantly (Figure 6B and 6C). Although the splice MO was clearly effective in the inhibition of zebrafish vasculature, the window of effective concentration was, however, close to that of the off-target curling of the tail. We therefore show the clearer UTR MO data images in Figure 6D. The major trunk and tail vessels (DA and CV) were not disrupted. Co-injection of h*FKBPL* mRNA with the UTR and splice MO rescued the phenotype in ≤91% of splice MO (*P*=0.002) and 83% of UTR MO–injected embryos (*P*=0.01; Figure 6B and 6C). The rescue of vascular disruption was also obtained with either full length N or C terminal *hFKBPL* mRNA. Interestingly, the vascular disrupting effects of *fkbpl* UTR MO were not seen in the presence of a z*cd44* MO (Figure 6C), supporting the dependency of Fkbpl on Cd44. This suggests that zFkbpl is also secreted, exerting its effect in a CD44-dependant outside-in signaling that we have previously reported for human cells. No effect of z*cd44* MO was seen in any of the injected embryos, and CD44 UTR MO control served as negative control. The vasculature did not seem altered in 24-hpf injected embryos.

## Discussion

FKBPL is emerging as an important anticancer protein having intra- and extracellular roles in tumor and endothelial cells.^10,14^ Although FKBPL mediates its intracellular roles predominantly via its interaction with other proteins, facilitated by its conserved TPR domain,^9,11^ its antiangiogenic effects are mediated through secretion and inside out signaling via the CD44 pathway.^12^ FKBPL is a nonconventional secreted protein lacking the signal peptide domain associated with proteins secreted via the golgi-ER pathway.^17^ We have previously reported that the antimigratory activity of FKBPL is reversed in the presence of a blocking antibody targeted to its antiangiogenic site, highlighting the role of the secreted protein in migration and angiogenesis.^12^ Here we observe that FKBPL is mainly secreted from endothelial and fibroblast cell lines (10- to 15-fold higher than cancer or epithelial cells), the 2 cell types with angiogenic and wound healing functions^18,19^ Importantly, FKBPL secretion is downregulated under hypoxia, a principal angiogenic stimulus that upregulates a range of angiogenic cytokines.^20^ This hypoxia-mediated regulation of FKBPL occurs specifically at the secreted level as the intracellular protein and RNA levels remain unaffected, further supporting the role of extracellular FKBPL in the regulation of angiogenesis. Interestingly, although VEGF is the proximate central angiogenic factor to hypoxia, regulating other downstream angiogenic events factors, it did not have any effect on FKBPL’s intra- or extracellular levels. Other angiogenic cytokines, IL-8 and FGF, did not affect FKBPL levels either. This further strengthens our hypothesis that FKBPL is upstream or independent of these pathways.

Although the role of FKBPL on tumor angiogenesis has been extensively investigated; its endogenous role in physiological or developmental angiogenesis has not yet been elucidated. Here we interrogated this function by developing an *Fkbpl* KO mouse model. The *Fkbpl* allele was targeted at the exon 2 region, leading to the complete deletion of the *Fkbpl* open reading frame (ORF), resulting in successful generation of mice heterozygous for *Fkbpl*. Attempts at generation of homozygous *Fkbpl* mice using *Fkbpl*^+/neo^ or *Fkbpl*^+/−^ crosses were unsuccessful, suggesting that the homozygous *Fkbpl* KO is embryonically lethal, potentially because of its important and essential role in murine development. Other angiogenic regulators demonstrating embryonic lethality are VEGF-A,^21^ DLL4,^22^ the Notch pathway genes, and angiopoietin,^23^ whereas *bFGF*-null mice do not develop vascular defects, and inactivation of *PlGF* or *VEGF-B* genes does not result in any major development abnormalities.^24^ Nevertheless, modest overexpression of VEGF results in excessive angiogenesis in mice, leading to severe abnormalities and embryonic lethality.^25^ Using timed matings, we identified the *Fkbpl* KO–mediated embryonic lethality occurred at ≤E8.5. This corresponds with early vasculogenesis in the mouse embryo, which commences around E7.0^26^ when angioblasts entering the embryo aggregate to form major vessels.

Further analysis of both *Fkbpl* heterozygous strains for expression of FKBPL mRNA and secreted levels correlated with the genotype and confirmed the absence of dosage compensation in these mice. We therefore proceeded to characterize the embryonic development of *Fkbpl*^+/neo^ mice. In accordance with the qPCR results, confocal microscopy images of intersomatic blood vessels (at comparable level of the body axis) indicate that E11.5 *Fkbpl*^+/neo^ mice showed significantly reduced levels of FKBPL compared with E11.5 *Fkbpl*^+/+^. FKBPL was strongly expressed in cells present in the lumen of blood vessels resembling primitive nucleated erythroblasts.^27^ This would seem to be consistent with its function as an angiogenesis regulator. The fenestrated morphology of vessels with clustering of FKBPL^+^ cells in *Fkbpl*^+/neo^ embryo sections possibly indicate finer, easily distorted vessels, poor circulation, or a decreased number of FKBPL^+^ cells. The distinct morphology of the endothelial cells in *Fkbpl*^+/neo^ embryos is consistent with the FKBPL-mediated effects on the cytoskeleton and the actin-tubulin dynamics as previously observed.^12^ These abnormalities in the embryonic vasculature, however, did not affect subsequent development, and no abnormality was observed in the histology of the organs. However, possible effects on longevity and age-related abnormalities remain to be investigated. To further evaluate the role of FKBPL in angiogenesis, induced models of angiogenesis were used. Ex vivo, aortae excised from *Fkbpl*^+/+^ mice demonstrated ordered sprouting; however, this process appeared to be significantly enhanced in *Fkbpl*^+/neo^, and vessel sprouts were highly branched and numerous. The increase in sprouting in FKBPL-deficient vessels is most likely because of its effect on endothelial cell migration rather than enhanced endothelial cell proliferation; our previous studies clearly demonstrate that FKBPL or its peptide derivatives do not affect endothelial cell viability.^10^ The reversal of this enhanced sprouting obtained in *Fkbpl*^+/neo^ aortae treated with AD-01^10^ supported the specificity of its antiangiogenic activity. Furthermore, the additive effect of *Fkbpl*^+/neo^ vessel sprouting obtained with VEGF treatment supports the hypothesis that FKBPL-mediated effects are independent of the VEGF pathway.

In an in vivo sponge assay, the increased vessel recruitment observed in *Fkbpl*^+/neo^ and *Fkbpl*^+/−^ mice confirmed their proangiogenic phenotype in vivo. The heterozygote vessels in the sponges appeared leaky on visual quantification. Irregular and increased vessel number was also observed in the Lewis lung carcinoma tumors grown in *Fkbpl*^+/neo^ or *Fkbpl*^+/−^ mice. Clearly, stromal FKBPL appeared to play a regulatory role in tumor growth and vascular development in this model; tumors in *Fkbpl* heterozygote mice grew faster with an increase in tumor angiogenesis. The role of tumor microenvironment in the regulation of tumor growth is indeed critical and regulates key processes of angiogenesis, metastasis, and cancer stem cell differentiation and is one of the hallmarks of tumorigenesis.^28^

To understand the mechanism of angiogenesis and its role in development, we used the zebrafish (*Danio rerio*) model. The FKBPL homologue (30% similarity) in zebrafish showed a similar structure in the C-terminal TPR domains, characteristic of the immunophilin family.^11^ The inhibition of intersegmental vessel formation in the zebrafish embryos with AD-01 treatment correlates to its well-characterized antiangiogenic effects observed earlier,^10,12^ as well as in this study. The endogenous role of Fkbpl in the fish model evaluated using morpholinos targeting the *zfkbpl* splice junction and the UTR resulted in an impairment of dorsal longitudinal anastomotic vessels and intersegmental vessel formation rescued with coinjection of either full length or N or C terminal *hFKBPL* mRNA. This indicated the conservation of FKBPL function across species. Interestingly, this function was dependent on zCd44 as the *zfkbpl* MO-mediated vessel impairment was abrogated in the *zcd44* morphant zebrafish embryos. This confirms our earlier studies on the CD44-dependent effect of FKBPL on migration of tumor and endothelial cells,^10,12^ further strengthening the role of zCd44 as zFkbpl’s extracellular membrane target. The disruption in the vessels suggests that complete loss of zFkbpl inhibits vessel formation or perhaps formation of nonrobust vessels because of loss of its antiangiogenic activity. Indeed, tight regulation of VEGF is essential for a functional vasculature and development; zebrafish studies with Vegf overexpression, as well as *zvegf* morphant embryos, have reported defective vasculature, resulting in pericardial edema and abnormal blood cell accumulation.^29^ Alternatively, the complete loss of FKBPL, as well as excess FKBPL (overexpression and exogenous addition^10,12^), may have inhibitory effects on angiogenesis. Dosage-dependant effects have been observed for other regulators of angiogenesis. For example, loss of one allele of DLL4 results in excessive and nonproductive angiogenesis,^30^ whereas *Dll4* overexpressing and *Dll4* KO mice both show impaired wound healing and antiangiogenic effects.^31^ Similar effects have been reported with focal adhesion kinase–deficient mice, where loss of one allele promoted angiogenesis and loss of both alleles inhibited it.^32^ Similarly, RGD (arginylglycylaspartic acid) mimetics that target integrins show differential dosage-dependent effects on angiogenesis.^33^ Further studies using conditional *Fkbpl^−/−^* mice to allow both the spatial and temporal ablation of FKBPL expression will be required to further elucidate the effect of FKBPL dosage.

Although the data presented here strongly suggest a role for FKBPL as a potent antiangiogenic mediator, our previous data also supports a role for FKBPL as an intracellular regulator of estrogen receptor signaling through its association with Hsp90.^13^ Therefore, the FKBPL-mediated effects on the embryonic lethality, the differences in our mouse and zebrafish data and perhaps why tumor growth rates in our FKBPL-deficient mice are higher, might also suggest a role for FKBPL-mediated regulation of estrogen receptor signaling. Indeed, the regulation of angiogenesis by estrogens and signaling via the Notch pathway has been previously reported.^34^ However, this is beyond the scope of the current article and would need to be further evaluated in future studies.

In conclusion, we have identified an essential role for FKBPL in murine embryonic and zebrafish vascular development. Loss of one *Fkbpl* allele in mice gives rise to a strong proangiogenic phenotype. Thus, deregulation of FKBPL levels could have serious implications for diseases associated with aberrant angiogenesis, widening the scope for the FKBPL-based clinical candidate, ALM201.

## Acknowledgments

We thank the staff at the Biological Resource Unit (BRU) for their help and support for all in vivo experiments, QUB, The Northern Ireland-Molecular Pathology Laboratory (NI-MPL), QUB for their help with the processing and scanning of tissue sections, and Ann Marie Higgins for her assistance with the vessel quantification during her MPharm level 4 project at the School of Pharmacy, QUB.

## Sources of Funding

This project was supported through a Biotechnology and Biological Sciences Research Council grant awarded to T. Robson (BB/I006958/1) covering salaries for A. Yakkundi, I. Hernández-Negrete, O. Lyubomska, M. Hanna. R. Bennett was supported by a Department of Employment and Learning-PhD studentship. The Northern Ireland-Molecular Pathology Laboratory is supported by Cancer Research UK, the Experimental Cancer Medicine Centre Network, and the Friends of the Cancer Centre.

## Disclosures

None.

## Significance

Secreted FK506-binding protein like (FKBPL) and its peptide derivatives inhibit cell migration and tumor angiogenesis by targeting CD44. We report that secretion of FKBPL is downregulated under the proangiogenic stimulus of hypoxia, independent of VEGF pathway. Using knockout mice and zebrafish models, we have established that FKBPL is essential for early embryonic development. Moreover, it is a critical regulator of angiogenic process; loss of one *Fkbpl* allele results in enhanced angiogenesis in experimental and tumor disease models. Modulation of FKBPL levels can therefore be exploited for therapeutic purposes. Reduced FKBPL levels, leading to enhanced angiogenesis, implicates a possible role in diseases other than cancer, such as cardiovascular and wound healing disease, thus extending the therapeutic utility of targeting FKBPL. FKBPL also has potential as a biomarker of angiogenesis and its secreted levels, offering ease of detection. This study therefore provides further support in enhancing the therapeutic and biomarker potential of FKBPL.
